# Life Satisfaction and Risk of Dementia: Evidence From Two Prospective Samples

**DOI:** 10.1093/geroni/igaf122.128

**Published:** 2025-12-31

**Authors:** Selin Karakose, Martina Luchetti, Yannick Stephan, Angelina R Sutin, Antonio Terracciano

**Affiliations:** Florida State University, Tallahassee, Florida, United States; Florida State University College of Medicine, Tallahassee, Florida, United States; University of Montpellier, Montpellier, Languedoc-Roussillon, France; Florida State University College of Medicine, Tallahassee, Florida, United States; Florida State University, Tallahassee, Florida, United States

## Abstract

Life satisfaction predicts better physical and mental health outcomes. There is promising evidence that its positive contribution may extend to healthier cognitive outcomes, including incident dementia. Samples from more countries are needed to evaluate the replicability and robustness of this association. This study examines the association between life satisfaction and dementia in two large, prospective, population-based cohorts from 15 European countries. Data from the English Longitudinal Study of Ageing (ELSA: N = 6979, 56.1% female; Mean age=65.35; Follow-up mean=11.27 years; 498 incident dementia) and the Survey of Health and Ageing and Retirement in Europe (SHARE: N = 24119, 55.5% female; Mean age=64.50; Follow-up mean: 10.90 years; 1655 incident dementia) were analyzed using Cox proportional hazards regression. In both samples, higher life satisfaction was associated with about 20% lower risk of dementia, an association that remained significant accounting for demographic (age, sex, race, education, marital status, and presence of children), psychological (depression), behavioral (physical activity and smoking), and clinical (obesity, diabetes, hypertension) risk factors. The association between life satisfaction and incident dementia was not moderated by sex, education, race, marital status, presence of children, or depressive symptoms in either sample. The associations were similar when the sample was restricted to participants 65 years and older and when participants who developed dementia within 5 years were excluded. The findings suggest that life satisfaction is a potential intervention target to reduce dementia risk and support healthier cognitive aging.

